# Unique Magnetic Resonance Imaging Findings in Opsoclonus-Myoclonus Syndrome Secondary to the West Nile Virus

**DOI:** 10.7759/cureus.67932

**Published:** 2024-08-27

**Authors:** James C Yang, Sepehr Zekavaty, Ryan D Rossi, Shamseldeen Y Mahmoud

**Affiliations:** 1 Radiology, Saint Louis University School of Medicine, Saint Louis, USA

**Keywords:** novel presentation, encephalitis, opsoclonus myoclonus syndrome, west nile virus, magnetic resonance imaging

## Abstract

Opsoclonus-Myoclonus syndrome is a rare neurological disorder that presents with oculomotor dysfunction and is associated with immunological triggers such as an infection. We present a patient with Opsoclonus-Myoclonus syndrome secondary to a West Nile virus (WNV) infection and focus on a unique series of magnetic resonance imaging findings. The following is a case report based on experience taking care of the patient as a member of the primary team in the hospital, chart review, and imaging findings obtained and reported through the department of radiology. A 61-year-old male presented with fatigue, ataxia, dysarthria, and fever after a recent cabin visit in the summer. The initial workup ruled out meningitis and stroke. The patient's condition deteriorated despite empiric treatment. Repeat magnetic resonance imaging (MRI) revealed patchy fluid-attenuated inversion recovery (FLAIR) hyperintensities in the cerebellar hemispheres. Further evaluation confirmed West Nile virus infection through positive immunoglobulin M (IgM) and immunoglobulin G (IgG) antibodies. This case underscores the importance of neuroimaging in evaluating encephalopathy, especially in the presence of multiple comorbidities. These findings contribute to the broader knowledge of West Nile virus encephalitis.

## Introduction

Opsoclonus-Myoclonus syndrome (OMS) is a rare disorder with clinical features including high-frequency oscillations of the eyes, ataxia, encephalopathy, behavioral changes, sleep changes, and myoclonus [1]. Precipitated by immunological factors, etiologies of OMS are usually paraneoplastic or idiopathic, but it can also be due to infections, drug side effects, and systemic diseases, such as AIDS or sarcoidosis [2]. Although a specific micro-organism leading to OMS is seldom identified, it has been reported to occur after infections with influenza, enteroviruses, varicella, human immunodeficiency virus, human herpesvirus 6, Coxsackie, Epstein-Barr virus, mumps, West Nile virus (WNV), Borrelia, and Streptococcus [3,4]. Even with an uncommon presentation of 2406 cases in 2023 in the United States (US), according to the Department of Health and Human Services, WNV remains the most common mosquito-borne disease in the US, with a mortality rate close to 10% [5]. In this case report, we present novel magnetic resonance imaging (MRI) findings of OMS secondary to WNV. 

## Case presentation

In the summer of 2023, a previously healthy 61-year-old male with multiple comorbidities, including hypertension, hyperlipidemia, type 2 diabetes, and tobacco use, presented with fatigue, dizziness, gait ataxia, dysarthria, and diplopia. The patient’s symptoms started after a cabin visit with his family in southern Missouri two weeks prior. He reported exposure to ticks. The physical exam was notable for dysarthria, tremors in outstretched hands, and rotatory nystagmus. There were no signs of any focal neurological deficits other than the symptoms described. The patient’s vitals were notable for hypertension (194 systolic/95 diastolic mm Hg) and a fever of 103.1 F. With medical treatment, the patient's blood pressure was labile but ranged between 130 systolic and 160 systolic, over 80 diastolic to 90 diastolic mm Hg.

**Figure 1:**
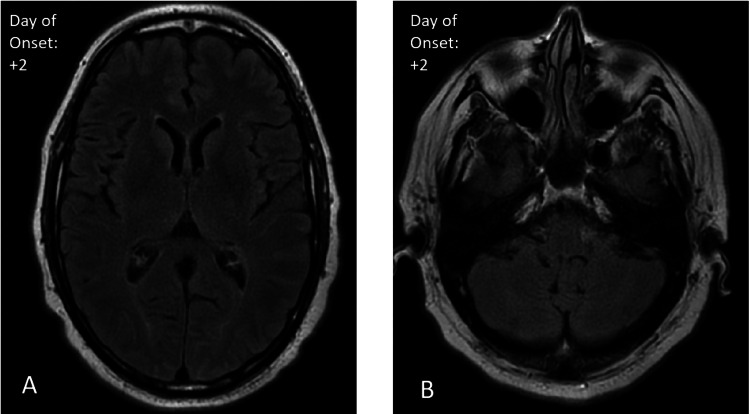
Baseline axial T2 FLAIR MRI. Day two after the onset of symptoms shows normal brain morphology in the cerebrum (A) and the cerebellum (B). There is no evidence of acute ischemia, hemorrhage, edema, or an abnormal mass effect. The brain volume and ventricular system are within normal limits for the patient's age. FLAIR: fluid-attenuated inversion recovery.

A workup for both sepsis and acute stroke was initiated. Serology was notable for leukocytosis. Head computed tomography (CT) was negative for acute hemorrhagic stroke. The initial MRI on day two was negative for intracranial abnormalities. There were no T2 fluid-attenuated inversion recovery (FLAIR) abnormalities in the cerebrum (Figure 1A) or cerebellum (Figure 1B). Cerebrospinal fluid (CSF) from a lumbar puncture showed an elevated protein level of 81 and a glucose level of 42. The electroencephalogram (EEG) did not identify any epileptic discharges. The patient received doses of methylprednisolone, acyclovir, vancomycin, and ceftriaxone without any improvement in symptoms. 

**Figure 2:**
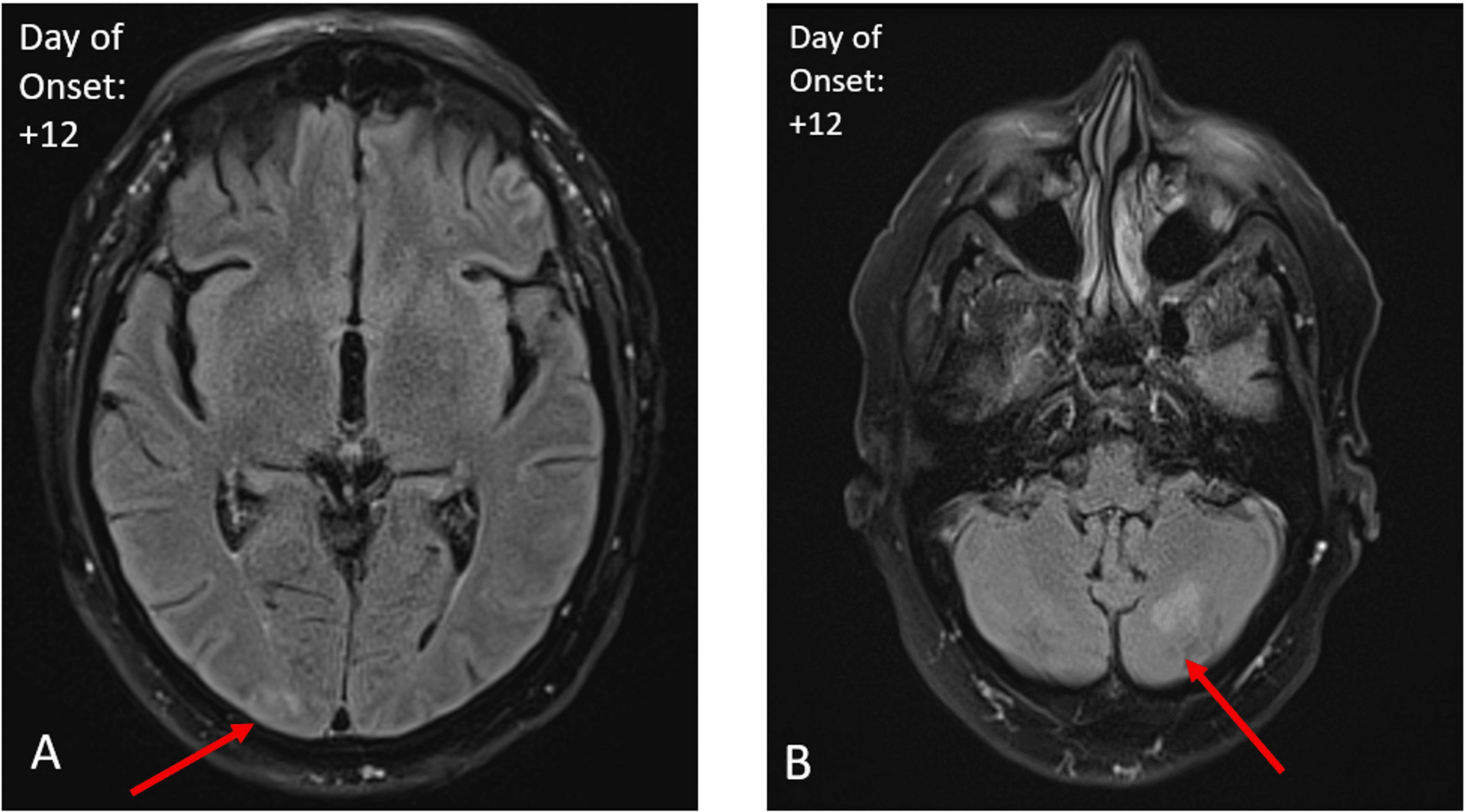
Follow-up axial fat-suppressed T2 FLAIR MRI. Day 12 after the onset of symptoms demonstrating mild sulcal FLAIR hyperintensity in the right occipital lobe (A) and patchy FLAIR hyperintensity in the left cerebellar hemisphere (red arrows) (B). FLAIR: fluid-attenuated inversion recovery.

**Figure 3:**
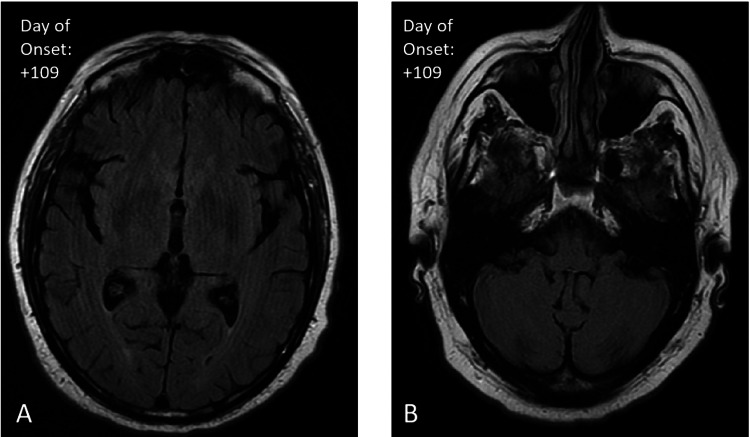
Post-treatment axial T2 FLAIR MRI. Day 109 after the onset of symptoms showing complete resolution of the previously observed right occipital (A) and left cerebellar abnormalities (B). FLAIR: fluid-attenuated inversion recovery.

All initial infectious serological and cerebrospinal workups, including West Nile polymerase chain reaction (PCR), were negative. However, West Nile immunoglobulin M (IgM) was 6.63 (reference range <0.89) and West Nile immunoglobulin G (IgG) was 3.68 (reference range <6.63), confirming a diagnosis of a West Nile virus infection (Table 1). Further paraneoplastic and autoimmune workups were negative. Repeat MRI 10 days after the initial MRI was notable for a subtle lack of sulcal FLAIR signal suppression along the right occipital lobe (Figure 2A) and patchy FLAIR hyperintensities in the left cerebellar hemisphere (Figure 2B). The patient’s symptoms persisted with somnolence, waxing and waning cognition, neurological symptoms consistent with OMS, and poor oral intake. The patient received intravenous immunoglobulin for five days with improvement in neurological symptoms and mentation. The patient received supportive care and was discharged after 22 days of hospitalization to a rehabilitation facility. Upon follow-up with neurology three months after discharge, the patient reported that he had returned to baseline, with only a residual mild intermittent right-hand tremor with brief myoclonus while writing. A follow-up MRI four months after discharge revealed complete resolution of the previously seen right occipital (Figure 3A) and left cerebellar abnormalities (Figure 3B). 

**Table 1:** A summary of notable labs with reference ranges included. Abnormal values are bolded. CSF: cerebrospinal fluid, PCR: polymerase chain reaction, IgG: immunoglobulin G, IgM: immunoglobulin M, L: liter, mL: milliliter, dL: deciliter, mg: milligram, nmol: nanomol.

Lab	Normal range	Patient’s value
Serum white blood cell count	4.4-10.7 x 10^9^/L	17.0 x 10^9^/L
Glucose (CSF)	40-70 mg/dL	42 mg/dL
Protein (CSF)	15-40 mg/dL	81 mg/dL
Total nucleated cells (CSF)	0-5 x 10^6^/L	47 x 10^6^/L
Red blood cells (CSF)	0-5 x 10^6^/L	70 x 10^6^/L
John Cunningham virus PCR (CSF)	Negative	Negative
Culture + gram stain (CSF)	Negative	Negative
West Nile virus PCR (CSF)	Not detected	Not detected
Herpes Simplex 1 + 2 PCR (CSF)	Not detected	Not detected
Acetylcholine binding antibody panel (blood)	0.00-0.25 nmol/L	<0.03 nmol/L
N-methyl-D-aspartate receptor antibody (blood)	<1:10	<1:10
Culture (blood)	Negative	Negative
Rocky Mountain spotted fever IgG antibody (blood)	Negative	Negative
Rocky Mountain spotted fever IgM antibody (blood)	0.00-0.89	0.57
*Ehrlichia chaffeensis* PCR (blood)	Negative	Negative
West Nile virus antibody IgG panel (blood)	<1.30	3.68
West Nile Virus antibody IgM panel (blood)	<0.9	6.63
Antinuclear antibody blood screen (blood)	Negative	Negative
Myeloperoxidase antibody (blood)	0.0-0.9 units	<0.2
Proteinase 3 antibody (blood)	0.0-0.9 units	<0.2
Cytoplasmic anti-neutrophil antibody titer (blood)	<1:20 titer	<1:20
Peri-nuclear anti-neutrophil antibody titer (blood)	<1:20 titer	<1:20
Lyme disease PCR (blood)	Negative	Negative
Human immunodeficiency virus antigen/antibody 1 & 2 (blood)	Non-reactive	Non-reactive
*Francisella tularensis* antibody IgG (blood)	<10 Units /mL	1 Unit
*Anaplasma phagocytophilum* antibody IgG (blood)	<1:80	<1:80

## Discussion

This case presentation is noteworthy because of the extensive differential diagnosis. The patient had multiple risk factors for stroke, including hypertension, hyperlipidemia, type 2 diabetes, and tobacco use. The abnormal MRI findings could also be interpreted as posterior reversible encephalopathy syndrome (PRES) in the setting of hypertension due to its posterior location and corresponding neurological findings, which cannot be ruled out. His presentation was also concerning for meningitis due to altered mental status, a fever, and leukocytosis. Seizure activity is also possible with a presentation such as this. Other less likely causes of these findings include autoimmune encephalitis and tick-borne encephalitis, given the patient’s recent exposure. However, the presence of elevated West Nile virus IgG and IgM (Table 1) despite a negative West Nile virus PCR is diagnostic for West Nile virus, as their sensitivity and specificity for the disease are consistently around 95% [6].

MRI findings can point to a specific etiology based on patterns, anatomical location, and signal characteristics of the lesions in many disease entities. For example, classic findings in herpes simplex virus encephalitis are characterized by asymmetric T2-FLAIR hyperintense lesions localized to the medial temporal lobes. Findings in West Nile virus are not as specific. However, reports describe T2-FLAIR hyperintensities in the deep gray matter of the brain, including the basal ganglia and thalamus [7]. This case presentation is novel because, to our knowledge, there are no previous reports of cerebellar T2-FLAIR hyperintensities found in WNV encephalitis presenting with OMS symptoms in this specific pattern in the literature, with a detailed series of three MRIs throughout the course of the disease from presentation to resolution [4,8,9]. Furthermore, the localization of the lesion to the cerebellum serves as a strong connection to the deficits described in this patient, as the cerebellum is responsible for the coordination of movement, maintaining posture, gait, and fine motor control [10].

The unique findings of this case also elucidate the unique pathophysiology behind WNV encephalitis. Due to the worsening clinical presentation of our patient, a repeat MRI was indicated, which revealed the abnormality 10 days after the original normal MRI. The other cases of OMS secondary to WNV did not have repeat MRI imaging, which suggests a specific temporal window between the onset of symptoms and when T2-FLAIR hyperintensities are visible on MRI. Literature suggests autoimmune versus vascular infiltration as possible mechanisms behind the pathophysiology of WNV encephalitis [11]. T2 FLAIR hyperintensity is nonspecific but could represent edema, demyelination, ischemia, or inflammation [12]. Lastly, follow-up MRI imaging revealed resolution with no residual abnormality, which sheds light on the prognosis and recovery process from OMS secondary to WNV, which can be fatal in many cases.

Ultimately, there is a great need for a West Nile virus MRI registry [13]. Commonly used databases by trainees and clinicians, such as Radiopaedia, currently have examples of MRI findings seen in WNV [14]. However, increasing the number of cases can be useful for radiologists. The antibody and PCR from the cerebrospinal fluid take several days to yield results; therefore, extracting as much information as possible from imaging can potentially lead to faster diagnosis and improved patient outcomes.

## Conclusions

Understanding different presentations of WNV encephalitis and the characteristic imaging findings is useful for clinicians to reach the correct diagnosis in the setting of encephalopathy. Parenchymal changes associated with WNV can fully recover and could be missed if imaging is delayed. Knowledge of the imaging appearance of rare pathologies, such as WNV, will assist radiologists in making the correct diagnosis.
